# Intestinal Indigestion

**Published:** 1884-09-20

**Authors:** Louis Starr

**Affiliations:** Lecturer on Diseases of Children in the Post-Graduate Course of the University of Pennsylvania


### INTESTINAL INDIGESTION.

BY LOUIS STARR, M.D.,

Lecturer on Diseases of Children in the Post-Graduate Course of the University of Pennsylvania. A Clinical Lecture delivered at the Children's Hospital, Philadelphia. Reported by Wm. H. Morrison, M.D.

GENTLEMEN: The previous history of the little patient before you is very incomplete, since we can learn nothing except that she has been in bad health for some time. She is eight years old and sufficiently tall for her age, but her face and limbs are thin. The muscles, however, are firm, and the skin is only slightly deficient in softness and flexibility. Her general strength is fair. The cheeks, as you will notice, have a moderately good, red color, and there are no dark rings around the eyes, although, if you will look closely, you will see that the conjunctivæ have a distinctly yellow tinge. Her tongue is perfectly healthy, the papillæ normal and the mucous membrane of the mouth healthy. Her appetite is good, there is no increased thirst, and she has neither vomiting nor eructations of flatus or sour liquid. She does not complain of abdominal pain, and the bowels are said to be open daily; but I am inclined to doubt this statement, for the belly is very greatly distended, and you will at once be struck with the contrast which this part of the body bears to the rest. While thus markedly altered in size, the abdomen is painless on palpation, and percussion elicits a tympanitic sound, showing that the enlargement is due to gaseous distension, and not to the presence of a solid tumor. The hepatic and splenic dullness are not increased. The heart and lungs are free from disease, there is no alteration in the pulse or surface temperature, and the urine is voided freely.

Now, what conclusions can be drawn from the points elicited in this case? In the first place, we can say positively that there is no gastric disorder, for the tongue is perfectly clean, the appetite good, and neither eructation, increased thirst, nausea nor vomiting is complained of. Still, the only symptom presented, viz., the uniform, great gaseous distension of the abdomen, indicates deranged digestion; consequently, we must look to the intestinal canal or its accessories for an explanation of the trouble. As already indicated, it is difficult to account for the absence of abdominal pain and disordered action of the bowels under the circumstances; but, since these symptoms are denied by the patient, let us see if the meteorism can be referred to any other cause than simple intestinal indigestion. The abdomen is uniformly distended, is painless on palpation, there are no indications of a tumor and very little constitutional disturbance, so that we may at once put intestinal obstruction, from intussusception or fecal accumulation for instance, out of the question. Sometimes in ataxic cases the muscular coat of the intestine, sympathizing in the general debility, ceases to afford the normal resistance to the contained gases, and these, expanding, greatly distend the gut; but this cause cannot be acting here, for the patient is but little below par. Caseation of the mesenteric glands (tabes mesenterica), especially when complicated, as it frequently is, with ulceration of the bowels, has meteorism for one of its symptoms; but there is, at the same time, diarrhœa and all the signs of chronic interference with the nutritive processes. Under such conditions the child wastes and grows pale and feeble, the face looks haggard, the sleep is disturbed, the appetite is capricious and thirst increased. Dilatation of the abdominal veins is often noticed, and occasionally edema of the feet and legs is met with, while the discovery of an irregularly shaped, slightly movable tumor in the umbilical region makes the diagnosis certain. These symptoms are entirely different from those presented by this case.

We are entirely justified, then, in attributing the meteorism to intestinal dyspepsia, which is the most common cause of abdominal distension in children.

Let us next study the manner in which the symptom is produced. There are no indications of impaired gastric function, and I think that we can assume that, as far as the stomach is concerned, the work of digestion is well done; but you know that only the albuminous articles of the diet are digested in the stomach, that a part even of this class of food passes the pylorus unaltered, and that the starches and fats are unaffected by the gastric secretion. In the intestine, therefore, some of the albumen and all of the starch and fat of a meal must be digested. This is mainly accomplished by two secretions poured into the upper part of the duodenum—namely, the bile and the pancreatic juice. Of these, the first takes the lesser part, merely assisting the gastric digestion and helping to emulsify the fats, the bulk of the work falling on the second.

The pancreatic juice contains four ferments: *a*, trypsin, which converts albuminous matter into peptones; *b*, curdling ferment, which curdles the casein of the milk; *c*, pancreatic diastase, which changes starch into sugar and dextrine; and *d*, emulsive ferment, which emulsifies and partly saponifies the fats. The major part of digestion is, therefore, accomplished in the intestine, and the pancreatic secretion is the most powerful and important agent; consequently, if this gland is at fault, if its secretion is diminished in quantity or poor in quality, more or less of each meal will remain in the intestine undigested, notwithstanding the fact that gastric digestion may be perfect.

You know, from a former lecture, that this undigested food, lying like a foreign body in the gut, irritates the delicate mucous membrane, causing catarrh with its uniform result, hypersecretion of mucous, and, from what has just been stated, you will infer that starch is one of the substances most likely to be imperfectly digested. There is present, then, a fermentable substance—starch, a ferment, mucous, and one of the encouraging conditions of fermentation, an elevated temperature. Of course, but one result can be expected; fermentation is set up, carbonic acid gas is liberated, and the intestine becomes distended.

As this explanation implies a catarrh of the intestinal mucous membrane, it would be interesting and satisfactory if we could find some proof of the existence of this condition in our patient. Constipation and the presence of mucous in the fecal evacuations, the common pathognomic symptoms, are wanting; but look again at the eyes; the conjunctivæ are, as you observe, quite yellow, indicating a slight degree of jaundice. Now jaundice, both in children and in adults, is most frequently catarrhal in its origin. In other words, it is due to more or less complete obstruction of the common bile duct by catarrhal swelling of the mucous membrane with the accompanying increased production of mucous, a lesion which is usually an *extension* of a pre-existing catarrh of the duodenal mucous membrane. Here, then, is the evidence which I think establishes the diagnosis.

For the successful management of this case a careful regulation of the diet is important. The starches and fats must be excluded, because they are digested in the intestine, and it is this portion of the digestive tract which we have found to be at fault. The starches are also unsuitable on account of their liability to undergo fermentation with the production of gas and a consequent increase of the abdominal distension. Three meals a day of the following articles of food should be taken: For breakfast, at 7:30 a. m., a bit of fresh fish or the lean of a mutton chop or a piece of tender beefsteak, with milk (either warmed or not, according to taste) and a single thin slice of stale bread, without butter. For dinner, at 2 p. m., the soft part of half a dozen oysters, a bowl of meat broth, entirely free from fat, or instead of this a piece of lean beefsteak, roast mutton or beef, a little spinach or well boiled cauliflower tops, and not more than a single slice of thin, unbuttered bread. For supper, at 7 o'clock in the evening, one or more glasses of milk, with a single slice of unbuttered bread. For drink she must take nothing but filtered water.

The medicinal treatment must be directed to the improvement of the impaired intestinal digestion, and as this has been traced to an inactive pancreatic secretion, we must endeavor to artificially supply the deficiency, just as in cases of stomach dyspepsia due to alterations in the gastric secretion we administer with the food pepsin and muriatic acid or supply an artificial gastric juice. Within the past few years a number of preparations have been introduced, purporting to contain the active principles of the pancreatic juice. One of these, Fairchild's Extractum Pancreatis, I have lately used extensively, and, so far, with very satisfactory results. To obtain these results, however, several things must be borne in mind: first, that the normal pancreatic juice is alkaline in reaction and that acids greatly impair, if they do not actually destroy, the activity of its ferments; second, that in health the pancreas throws out its secretion most freely from two to three hours after a meal has been swallowed, or about the time that the food is passing from the stomach into the small intestine; third, that at this time the contents of the stomach are still acid in reaction. These facts show us that, to be of any service, the Extractum Pancreatis must be given at the proper time, two and a half hours after taking food, and must be safely conducted through the stomach, a feat accomplished only by guarding it with a full dose of an alkali, as bicarbonate of sodium.

The proper dose for a child of the age of our patient is two and a half grains. I shall order the following prescription:

℞ Extract pancreatis........................gr. xxx,
Sodii bicarbonate..........................ʒ j.
M. et ft., chart No. xij.

Sig. One powder to be taken two and a half hours after each meal.

Nux vomica is also indicated, partly to give tone to the muscles of the intestine, which must be in some degree weakened by the constant distension, and partly to encourage proper glandular action. I shall, therefore, order three drops of tincture of nux vomica with a teaspoonful of compound infusion of gentian before each meal.

Finally, to assist in the reduction of the abdominal distension, it will be well to rub the belly thoroughly twice a day with a stimulating liniment, such as turpentine and olive oil, one part to three.—*Archives of Pedriatics.*

